# LINE-1 hypomethylation status of circulating cell-free DNA in plasma as a biomarker for colorectal cancer

**DOI:** 10.18632/oncotarget.14439

**Published:** 2017-01-02

**Authors:** Yuzo Nagai, Eiji Sunami, Yoko Yamamoto, Keisuke Hata, Satoshi Okada, Koji Murono, Koji Yasuda, Kensuke Otani, Takeshi Nishikawa, Toshiaki Tanaka, Tomomichi Kiyomatsu, Kazushige Kawai, Hiroaki Nozawa, Soichiro Ishihara, Dave S.B Hoon, Toshiaki Watanabe

**Affiliations:** ^1^ Department of Surgical Oncology, Faculty of Medicine, The University of Tokyo, Bunkyo-ku, Tokyo, Japan; ^2^ Department of Translational Molecular Medicine, Div. Molecular Oncology, John Wayne Cancer Institute, Saint John's Hospital and Health Center, Santa Monica, CA, USA

**Keywords:** colorectal cancer, cell-free DNA, LINE-1, hypomethylation, plasma

## Abstract

Colorectal cancer (CRC) is a serious public health problem and non-invasive biomarkers improving diagnosis or therapy are strongly required. Circulating cell-free DNA (cfDNA) has been a promising target for this purpose. In this study, we evaluated the potential of long interspersed nuclear element-1 (LINE-1) hypomethylation as a blood biomarker for CRC. LINE-1 hypomethylation level in plasma cfDNA in 114 CRC patients was retrospectively examined by absolute quantitative analysis of methylated alleles real-time PCR, and was expressed using LINE-1 hypomethylation index (LHI) [unmethylated copy number/ (methylated copy number + unmethylated copy number)]. Greater LHI values indicated enhanced hypomethylation. In our clinicopathological analysis, CRC patients with large tumors (≥6.0 cm), advanced N stage (≥2), and distant metastasis (M1) had statistically significantly higher cfDNA LHI than other CRC patients, suggesting cfDNA LHI as a disease progression biomarker for CRC. Furthermore, early stage I/II (n = 57) as well as advanced stage III/IV (n =57) CRC patients had significantly higher cfDNA LHI than healthy donors (n=53) [stage I/II: median 0.369 (95% confidence interval, 0.360–0.380) vs. 0.332 (0.325–0.339), *P* < 0.0001; stage III/IV: 0.372 (0.365–0.388) vs. 0.332 (0.325–0.339), *P* < 0.0001]. The receiver operating characteristic analysis showed that cfDNA LHI had the detection capacity of CRC with area under the curve(AUC) of 0.79 and 0.83 in stage I/II and stage III/IV CRC patients, respectively. The present study demonstrated for the first time the potential of plasma cfDNA LHI as a novel biomarker for CRC, particularly for early stage detection.

## INTRODUCTION

Colorectal cancer (CRC) is a serious public health problem third leading cause of cancer-related deaths worldwide, owing to its high incidence and cancer-related mortality [1]. A wide range of biomarkers, such as those for early detection, tumor progression, prediction of prognosis, and therapeutic monitoring have been investigated in the pursuit of the overall improvement of CRC patients’ outcomes.

In recent years, tumor-related circulating cell-free DNA (cfDNA) in plasma and serum has been a promising target for cancer biomarker studies. Clinical application of tumor-related cfDNA in plasma or serum has been termed “liquid biopsy” and utilized as a non-invasive method for the detection of tumor specific genetic and epigenetic alterations [2–5].

Aberrant DNA hypomethylation is one of the major DNA methylation abnormalities in cancer. It generally occurs in repetitive transposable DNA elements such as long interspersed nuclear element-1 (LINE-1) as well as short interspersed nucleotide elements (SINE or ALU), and is associated with genomic instability [6]. In particular, highly repetitive sequences of non-coding genomic LINE-1 retrotransposon comprise approximately 17–18% of the human genome, therefore, the methylation status of LINE-1 is considered to be an excellent indicator of the global DNA methylation status [7]. Many epigenetic studies have reported the LINE-1 hypomethylation in various cancers including CRC [8–12], breast cancer [13], gastric cancer [14, 15], melanoma [16], and hepatocellular carcinoma (HCC) [17]. In fact, previous studies have reported a correlation between the presence of LINE-1 hypomethylation in CRC tissue with tumor progression [12] and poor prognosis [8, 9, 11, 18, 19].

Based on the data presented by Hoshimoto *et al*., increased levels of LINE-1 hypomethylation were observed in the serum cfDNA of stage III or IV malignant melanoma patients, compared to healthy donors [16]. Nonetheless, the LINE-1 hypomethylation status in plasma or serum cfDNA from CRC patients has not been further investigated. In this study, using absolute quantitative analysis of methylated alleles (AQAMA) real-time PCR method, we quantified LINE-1 hypomethylation level in plasma cfDNA from 114 CRC patients, and analyzed the association with various clinicopathological factors. Moreover, based on our previous finding of LINE-1 hypomethylation manifestation at very early stages of CRC development [12], we hypothesized that LINE-1 hypomethylation can be a novel biomarker for early CRC detection. To that end, we evaluated the potential of LINE-1 hypomethylation in plasma cfDNA as a blood biomarker for early stage CRC detection.

## RESULTS

### Association between clinicopathological factors, cfDNA concentration and cfDNA LHI in CRC patients

Plasma cfDNA concentration and cfDNA LINE-1 hypomethylation levels in 114 CRC patients who underwent surgical resection at The University of Tokyo Hospital between April 2012 and June 2014 were analyzed. The LINE-1 hypomethylation levels were evaluated by a modified absolute quantitative analysis of methylated alleles (AQAMA) real-time PCR assay, which were validated in our previous studies [12, 13, 16, 20], and was expressed as LINE-1 hypomethylation index (LHI); greater LHI indicated enhanced hypomethylation.

Table 1 exhibits the associations between clinico-pathological features and both cfDNA concentration and cfDNA LHI. For cfDNA concentration, CRC patients with distant metastasis (M1) had significantly higher cfDNA concentration than those without distant metastasis (M0) [11.7 (11.1–15.1) vs. 10.2 (8.8–11.5) ng/mL, *P* = 0.03]. No statistically significant correlations were found between cfDNA concentration and tumor size, tumor location, tumor differentiation, lymphatic invasion, venous invasion, preoperative carcinoembryonic antigen (CEA) and carbohydrate antigen 19-9 (CA 19-9) levels, T stage and N stage.

**Table 1:** Clinicopathological factors and cfDNA concentration and cfDNA LHI in CRC patients (n = 114)

Factors	Number (%)	cfDNA concentration, median (95% CI), ng/mL	P value	cfDNA LHI, median (95% CI)	P value
Tumor size			0.64		0.04
<6.0 cm	85 (74.6%)	10.7 (9.6–11.7)		0.368 (0.362–0.374)	
≥6.0 cm	29 (25.4%)	11.1 (9.1–14.5)		0.385 (0.365–0.407)	
Location			0.08		0.77
Right-side	34 (29.8%)	11.8 (9.8–16.6)		0.372 (0.36–0.388)	
Left-side	80 (70.2%)	10.4 (8.9–11.4)		0.371 (0.362–0.382)	
Tumor differentiation			0.75		0.52
WD	46 (40.4%)	10.9 (8.9–11.9)		0.373 (0.362–0.382)	
MD	60 (52.6%)	11.2 (9.1–12.6)		0.369 (0.357–0.384)	
PD/Mucinous	8 (7.0%)	10.6 (8.2–37.5)		0.387 (0.350–0.440)	
Lymphatic invasion			0.97		0.54
Negative	69 (60.5%)	10.5 (9.6–11.7)		0.373 (0.365–0.383)	
Positive	45 (39.5%)	11.5 (8.4–12.6)		0.370 (0.352–0.377)	
Venous invasion			0.72		0.86
Negative	25 (21.9%)	10.4 (8.1–12.6)		0.372 (0.361–0.376)	
Positive	89 (78.1%)	11.1 (9.8–11.8)		0.371 (0.362–0.383)	
Preoperative CEA			0.17		0.57
<5.0 ng/mL	52 (45.6%)	10.3 (8.2–11.5)		0.368 (0.364–0.382)	
≥5.0 ng/mL	62 (54.4%)	11.3 (9.8–12.8)		0.373 (0.365–0.388)	
Preoperative CA19-9			0.28		0.49
<37 ng/mL	83 (72.8%)	10.4 (8.9–11.6)		0.371 (0.363–0.383)	
≥37 ng/mL	31 (27.2%)	11.4 (9.4–15.1)		0.372 (0.349–0.381)	
T stage			0.57		0.69
T1–2	28 (24.6%)	10.1 (8.1–11.6)		0.372 (0.363–0.382)	
T3–4	86 (75.4%)	11.2 (9.8–11.9)		0.371 (0.360–0.383)	
N stage			0.61		0.01
N0–1	90 (78.9%)	10.6 (9.4–11.5)		0.368 (0.357–0.373)	
N2–3	24 (21.1%)	11.6 (8.9–15.1)		0.389 (0.371–0.411)	
M stage			0.03		0.03
M0	87 (76.3%)	10.2 (8.8–11.5)		0.368 (0.360–0.373)	
M1	27 (23.7%)	11.7 (11.1–15.1)		0.388 (0.373–0.402)	

Abbreviations: cfDNA, circulating cell-free DNA; LHI, LINE-1 hypomethylation index; CRC, colorectal cancer; CI, confidence interval; WD, well differentiated; MD, moderately differentiated; PD, poorly differentiated; CEA, carcinoembryonic antigen; CA 19-9, carbohydrate antigen 19-9.

Although no correlation was observed between cfDNA LHI and T stage when looking at cfDNA LHI, patients with tumors larger than 6.0 cm had significantly higher cfDNA LHI than patients with tumors smaller than 6.0cm [0.385 (0.365–0.407) vs. 0.368 (0.362–0.374), *P* = 0.04]. In addition, patients with advanced N stage (≥2) and distant metastasis (M1) had significantly higher cfDNA LHI [N stage, 0.389 (0.371–0.411) vs. 0.368 (0.357–0.373), *P* = 0.01; M stage, 0.388 (0.373–0.402) vs. 0.368 (0.360–0.373), *P* = 0.03]. No correlation was found between cfDNA LHI and standard prognostic factors for CRC; tumor location, tumor differentiation, lymphatic invasion, venous invasion, and preoperative CEA and CA19-9 blood levels.

### Comparison of demographic factors between healthy donors and CRC patients

We compared demographic factors (sex, age, BMI, and smoking status) between healthy donors (n = 53) and CRC patients (n = 114). As shown in Table 2, CRC patients were significantly older (*P* < 0.0001) than healthy donors and had a slightly higher rate of current smokers (*P* = 0.07), suggesting that age and smoking status may be potential confounding factors in our cohort. However, stratification analysis showed that in both healthy and CRC patients, neither cfDNA concentration nor cfDNA LHI were associated with all demographic factors including age and smoking status (Table 3). Based on these results, we included all healthy donors and CRC patients into our subsequent analysis.

**Table 2:** Comparison of demographic factors between healthy donors and CRC patients

Factors	Healthy donors (n = 53)	CRC patients (n =114)	P value
Sex			0.38
Male	34 (64.1%)	65 (57.0%)	
Female	19 (35.9%)	49 (43.0%)	
Age	50.6 (SD 19.3)	63.0 (SD 12.5)	< 0.0001
BMI	22.5 (SD 2.5)	22.2 (SD 3.3)	0.43
Smoking status			0.07
Current	7 (13.2%)	29 (25.4%)	
Former/None	46 (86.8%)	85 (74.6%)	

Abbreviations: CRC, colorectal cancer; BMI, body mass index; SD, standard deviation.

**Table 3:** Demographic factors and cfDNA concentration and cfDNA LHI in healthy donors and CRC patients

Factors	Healthy donors (n =53)					CRC patients (n = 114)				
	Number (%)	cfDNA concentration	*P* value	cfDNA LHI	*P* value	Number (%)	cfDNA concentration	*P* value	cfDNA LHI	*P* value
Sex			0.32		0.32			0.97		0.11
Male	34 (64%)	8.0 (6.4–12.2)		0.331 (0.324–0.337)		65 (57%)	11.1 (9.1–11.7)		0.365 (0.357–0.374)	
Female	19 (36%)	7.7 (7.0–9.3)		0.334 (0.320–0.345)		49 (43%)	11.1 (8.9–12.8)		0.373 (0.370–0.391)	
Age			0.93		0.48			0.15		0.96
<60 years	34 (64%)	8.3 (4.9–10.4)		0.331 (0.315–0.344)		44 (39%)	9.7 (8.2–11.2)		0.372 (0.360–0.383)	
≥60 years	19 (36%)	7.2 (6.6–10.0)		0.334 (0.325–0.344)		70 (61%)	11.5 (10.0–12.7)		0.371 (0.362–0.381)	
BMI			0.80		0.52			0.51		0.27
<25	45 (85%)	7.7 (6.4–9.5)		0.334 (0.325–0.340)		89 (78%)	11.1 (9.8–11.5)		0.373 (0.365–0.383)	
≥25	8 (15%)	7.1 (6.6–23.9)		0.327 (0.316–0.355)		25 (22%)	11.7 (7.7–15.7)		0.367 (0.350–0.374)	
Smoking			0.23		0.30			0.48		0.41
Current	7 (13%)	10.6 (2.26–23.9)		0.326 (0.285–0.349)		29 (25%)	11.1 (7.7–12.6)		0.373 (0.360–0.389)	
Former/None	46 (87%)	7.6 (7.0–9.3)		0.334 (0.325–0.340)		85 (75%)	11.1 (9.8–11.9)		0.370 (0.362–0.376)	

Note: cfDNA concentration and cfDNA LHI were expressed as median (95% confidence interval).

Abbreviations: cfDNA, circulating cell-free DNA; LHI, LINE-1 hypomethylation index; CRC, colorectal cancer; BMI, body mass index.

### Comparison of cfDNA concentration and cfDNA LHI between healthy donors and CRC patients

**Figure 1:**
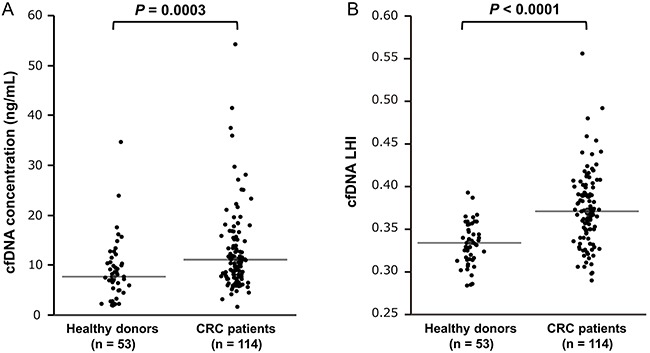
Comparison of cfDNA concentration and cfDNA LHI between healthy donors and CRC patients **A**. cfDNA concentration of 114 CRC patients was significantly higher than that of 53 healthy donors (Mann–Whitney U test, *P* = 0.0003). **B**. cfDNA LHI of 114 CRC patients was significantly higher than that of 53 healthy donors (Mann–Whitney U test, *P* < 0.0001). The horizontal line represents the median value.

Figure 1 shows that cfDNA concentration and cfDNA LHI of CRC patients were significantly higher than those of healthy donors [cfDNA concentration, 11.1 (9.8–11.6) vs. 7.7 (7.0–9.5) ng/mL, *P* = 0.0003, Figure 1A; cfDNA LHI, 0.371 (0.365–0.376) vs. 0.332 (0.325–0.339), *P* < 0.0001, Figure 1B].

**Figure 2:**
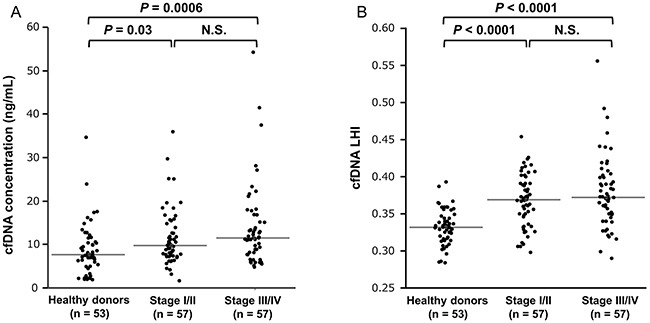
Comparison of cfDNA concentration and cfDNA LHI between healthy donors and early (stage I/II) and advanced (stage III/IV) CRC patients **A**. A nonparametric multiple comparison Steel-Dwass test showed that both early stage I/II and advanced stage III/IV CRC patients had significantly higher cfDNA concentration than healthy donors [stage I/II, 9.8 (8.6–11.5) vs. 7.7 (7.0–9.5) ng/mL, *P* = 0.03; stage III/IV, 11.5 (11.0–13.0) vs. 7.7 (7.0–9.5) ng/mL, *P* = 0.0006]. There was no statistically significant difference of cfDNA concentration between early and advanced CRC patients (*P* = 0.31). The horizontal line represents the median value. **B**. A nonparametric multiple comparison Steel-Dwass test showed that both early stage I/II and advanced stage III/IV CRC patients had significantly higher cfDNA LHI than healthy donors [stage I/II, 0.369 (0.360–0.380) vs. 0.332 (0.325–0.339), *P* < 0.0001; stage III/IV, 0.372 (0.365–0.388) vs. 0.332 (0.325–0.339), *P* < 0.0001]. There was no statistically significant difference of cfDNA LHI between early and advanced CRC patients (*P* = 0.66). The horizontal line represents the median value.

Next we stratified CRC patients into early (stage I/II, n = 57) and advanced (stage III/IV, n = 57) groups. As shown in Figure 2, early stage I/II CRC patients had significantly higher cfDNA concentration and cfDNA LHI than healthy donors (n=53) [cfDNA concentration, 9.8 (8.6–11.5) vs. 7.7 (7.0–9.5) ng/mL, *P* = 0.03, Figure 2A; cfDNA LHI, 0.369 (0.360–0.380) vs. 0.332 (0.325–0.339), *P* < 0.0001, Figure 2B]. Similarly, advanced stage III/IV CRC patients had significantly higher cfDNA concentration and cfDNA LHI [cfDNA concentration, 11.5 (11.0–13.0) vs. 7.7 (7.0–9.5) ng/mL, *P* = 0.0006, Figure 2A; cfDNA LHI, 0.372 (0.365–0.388) vs. 0.332 (0.325–0.339), *P* < 0.0001, Figure 2B] than healthy donors. On the other hand, there were no statistically significant differences in cfDNA concentrations and cfDNA LHI between early and advanced stage CRC patients (cfDNA concentration, *P* = 0.31, Figure 2A; cfDNA LHI, *P* = 0.66, Figure 2B).

### ROC curve analysis for the detection capacity of CRC

**Figure 3:**
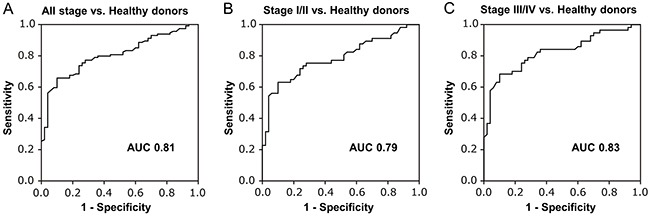
ROC curve analysis assessing the capacity of cfDNA LHI to distinguish CRC patients from healthy donors **A**. ROC curve analysis of cfDNA LHI for all stage CRC patients. Using the cut-off value of 0.360, cfDNA LHI could distinguish CRC patients with 65.8% sensitivity and 90.0% specificity (AUC 0.81, *P* < 0.0001). The optimal cut-off value was defined as the highest Youden index [(specificity + sensitivity) − 1]. **B**. ROC curve analysis for early CRC patients (stage I/II). cfDNA LHI showed sensitivity 63.2%, specificity 90.0% (AUC 0.79, *P* < 0.0001). **C**. ROC curve analysis for advanced CRC patients (stage III/IV). cfDNA LHI showed sensitivity 68.4%, specificity 90.0% (AUC 0.83, *P* < 0.0001).

We examined the capacity of cfDNA concentration and cfDNA LHI as a biomarker for distinguishing CRC patients from healthy donors using receiver operating characteristic (ROC) curve analysis. The optimal cut-off values were determined from the highest Youden index [21]. Using the cut-off value of 0.360, cfDNA LHI distinguished CRC patients with 65.8% sensitivity and 90.0% specificity (area under the curve (AUC) 0.81, *P <* 0.0001, Figure 3A). Next we performed subgroup analysis for early (stage I/II) and advanced (stage III/IV) CRC patients. cfDNA LHI distinguished early CRC patients with 63.2% sensitivity and 90.0% specificity (AUC 0.79, *P* < 0.0001, Figure 3B) and advanced CRC patients with 68.4% sensitivity and 90.0% specificity (AUC 0.83, *P* < 0.0001, Figure 3C).

Using the cut-off value of 10.7 ng/mL, cfDNA concentration distinguished early CRC patients with 42.1% sensitivity and 75.0% specificity (AUC 0.64, *P* = 0.03) and advanced CRC patients with 63.2% sensitivity and 75.0% specificity (AUC 0.70, *P* = 0.003), rendering it less adequate for CRC detection than cfDNA LHI.

### Comparison of sensitivity between CEA and cfDNA LHI for CRC detection

We assessed the capacity of a standard blood CEA biomarker for CRC detection in CRC patients. Table 4 shows that the sensitivity of CEA for CRC detection was 40.4% in stage I/II CRC with the conventional cut-off value of 5.0 ng/mL. On the other hand, the sensitivity of cfDNA LHI was 63.2% in stage I/II CRC, indicating its higher sensitivity for early CRC detection than CEA.

**Table 4:** Comparison of sensitivity between CEA and cfDNA LHI for CRC patients

	All stage (n = 114)	Stage I/II (n = 57)	Stage III/IV (n= 57)
CEA (≥5.0 ng/mL)			
Sensitivity	54.4% (62/114)	40.4% (23/57)	68.4% (39/57)
cfDNA LHI			
Sensitivity	65.8% (75/114)	63.2% (36/57)	68.4% (39/57)

Abbreviations: cfDNA, circulating cell-free DNA; LHI, LINE-1 hypomethylation index; CRC, colorectal cancer; CEA, carcinoembryonic antigen.

## DISCUSSION

In this study, we examined 114 plasma samples of CRC patients, and quantified LINE-1 hypomethylation status in plasma cfDNA by AQAMA PCR method. The efficacy of this assay has been validated in several previous studies [12, 13, 16, 20]. We confirmed that even early stage I/II as well as advanced stage III/IV CRC patients had significantly higher cfDNA LHI than healthy donors. Detection of early stage I/II CRC through cfDNA LHI was accomplished with 63.2% sensitivity and 90.0% specificity (AUC 0.79), suggesting the potential utility of cfDNA LHI as a blood biomarker for early CRC detection.

Colonoscopy is the gold standard for CRC diagnosis, given its more than 90% sensitivity and specificity [22]. However, due to its invasive nature and possible morbid complications such as bowel perforation, most patients are reluctant to undergo colonoscopy. At present, fecal occult blood test (FOBT) is the most frequently used non-invasive modality in CRC screening program in the USA. Prospective randomized controlled trials showed that FOBT screening reduced CRC-related mortality [23]. Furthermore, recent fecal immunochemical test (FIT) has shown high CRC detectability [24], while the utility of stool-based DNA assays has been reported as a new application for CRC diagnosis [25]. Nonetheless, FOBT has some limitations, such as its relatively low sensitivity for early-stage or proximal colon cancer [24, 26]. Moreover, adherence rate to the CRC screening program based on FOBT and colonoscopy remains low at around 50% [27, 28], and it is reported that many people who avoid current FOBT program prefer a simple blood-based test instead [29]. Our non-invasive blood-based method would be particularly beneficial for patients who are averse to stool-based test.

Most blood methylation biomarkers target a single chromosome region. As a result, the amount of target cfDNA fragments in blood circulation can be very limited particularly after bisulfite conversion [30], raising concerns about false negative results. To increase the assay sensitivity, a large amount of plasma is sometimes needed; commercially offered mSEPT9 test such as Epi proColon (Epigenomics AG, Berlin, Germany) requires 3.5 mL of plasma [31]. Conversely, the abundant number of LINE-1 copies in the human genome enabled us to quantify the absolute methylation level with 0.5 mL of plasma without any additional applications such as digital PCR or next generation sequencing. Moreover, our LINE-1 assay is particularly advantageous when using plasma, which is preferred due to its relatively low genomic DNA contamination, but has lower amount of cfDNA than serum [3].

Although the sensitivity and specificity of CEA are considered to be insufficient for CRC detection, CEA is the most routinely measured blood biomarker for CRC. Our data showed that cfDNA LHI had higher sensitivity for early stage I/II CRC than CEA. In a recent systematic review [32], the pooled specificity of CEA for CRC detection was 88.0% with the conventional cut-off value of 5.0 ng/mL, which is similar to our results’ specificity of 90.0%, suggesting cfDNA LHI to be more suitable for early CRC detection than CEA. Several previous studies have also reported the utility of simple quantitative assessment of cfDNA concentration for CRC diagnosis [33–36]. However, in our cohort, cfDNA concentration demonstrated lower detectability of CRC when compared to cfDNA LHI. Additionally, considering that cfDNA concentration can be affected by various non-malignant diseases and physiological conditions [3], its diagnostic utility for CRC is further discredited. We did not observe a statistically significant difference in cfDNA LHI and cfDNA concentrations between early (stage I/II) and advanced (stage III/IV) CRC patients. Although LINE-1 hypomethylation occurs at very early stage in CRC development [12], several studies have reported that LINE-1 hypomethylation status in CRC tissue was independent of CRC stage [8, 11, 37, 38]. Overall, LINE-1 hypomethylation is believed to remain relatively stable during CRC progression [39]. These findings may partially explain our results that no difference of cfDNA LHI was observed between early and advanced CRC patients. On the other hand, CRC patients with highly advanced stage (N ≥2 and M1) and large tumor size (≥6.0 cm) showed significantly higher cfDNA LHI than other patients in the study, implying the possibility of cfDNA LHI as a biomarker for advanced CRC progression and/or distant metastasis.

Four limitations of this study must be noted. First, LINE-1 hypomethylation in plasma cfDNA is not always specific to CRC. Similar to other methylation blood biomarkers, no CRC-specific DNA methylation biomarkers have been identified at this time. However, considering the recent high incidence and cancer-related mortality of CRC, it may be beneficial to employ our non-invasive blood assay and easily measure cfDNA as a first step towards CRC detection and recurrence after surgery. Repetitive analysis at specific intervals of cfDNA LHI may also be useful for disease monitoring as in early detection of recurrence following the surgical resection of CRC in high risk patients. Second, in comparison with healthy donors, we assessed four demographic factors (sex, age, BMI, and smoking factors) that may influence cfDNA LHI. However, DNA methylation can also be affected by other factors such as race, alcohol consumption, physical activity, and diet [40], whose associations with LINE-1 hypomethylation remain undetermined [40]. Third, during the median follow-up of 24.6 months, eight patients died of CRC (one stage II, one stage III and six stage IV) and 13 patients in stage I–III experienced tumor recurrence after the curative resection (four stage II and nine stage III). However, we could not detect a statistically significant correlation between patients’ prognosis and cfDNA LHI. This may be due to the short follow-up period. Finally, this was a prospective pilot study from a single institute small patient cohort. Therefore, prospective studies and multi-institutional validation are needed in the future.

In conclusion, the present study is the first report demonstrating the potential of LINE-1 hypomethylation in plasma cfDNA as a blood biomarker for CRC detection, particularly for early stages of the disease.

## MATERIALS AND METHODS

### Patients and healthy donors

We analyzed a total of 114 plasma samples obtained from consecutive CRC patients who underwent surgical tumor resection at the University of Tokyo Hospital between April 2012 and June 2014, whose plasma samples were available for analysis. Exclusion criteria for this study consisted of any patients with a history of malignant disease or colitis associated CRC, as well as patients receiving any preoperative treatments or radiotherapy. No other malignant tumors except CRC were found in patients after preoperative examinations. A detailed database of clinicopathological information was developed for statistical analysis. The cancer's histological grade and clinical stage were identified in accordance with the seventh edition of the TNM classification of the Union for International Cancer Control (UICC).

Fifty-three healthy donors were also analyzed. All healthy donors were having medical check-ups periodically and had no history of cancer or bowel symptoms, representing a normal Japanese adult population cohort at average risk of CRC. For the healthy donors > 50 yrs, prior to blood sample collection, colonoscopy examination was performed to confirm the absence of advanced adenoma or CRC.

The study protocol was approved by the ethics committee of the University of Tokyo Hospital and written informed consents were obtained from all participating patients.

### Specimen demographics

Prior to the operation, peripheral blood (10 mL) of each patient was collected into EDTA-containing blood tubes. Plasma was obtained by centrifugation of the whole blood samples at 1,500 g for 10 min and immediately stored at −80°C until analysis. Similarly, peripheral blood collection (5mL) of healthy donors was followed by extraction of plasma and storage as describe above.

### cfDNA extraction from plasma

Plasma samples were thawed and microcentrifuged at 4°C and 12,000 g for 3 min to remove cell debris. cfDNA was extracted from plasma as previously described [12, 16, 41]. In brief, aliquots of 500 μL plasma were diluted with 0.9% NaCl and mixed with a premix consisting of proteinase K and SDS. After incubation at 50°C for 3 h, cfDNA was treated with phenol–chloroform-isoamyl alcohol (25:24:1, pH 8.0; Nippon Gene) and precipitated with isopropanol.

### Measurement of cfDNA concentration

DNA concentration of each plasma sample was quantified using Varioskan Flash (ThermoFisher Scientific) and a Quani-iT PicoGreen dsDNA Assay Kit (Life Technologies) according to the manufacturer's instructions.

### Sodium bisulfite conversion

Sodium bisulfite conversion was performed as previously described [42]. In brief, up to 500 ng of DNA was denatured in 0.3 M NaOH at 37°C for 15 min and dissolved in a solution consisting of 3.06 M sodium bisulfite (Sigma-Aldrich) and 0.5 mM hydroquinone (Sigma-Aldrich) adjusted to pH 5.0 with NaOH. The solution was subjected to 15 cycles of denaturation at 95°C for 30 s and incubation at 50 °C for 15 min, desalted and desulfonated on a Zymo-Spin Column (Zymo Research), and eluted with 20 μL of Tris-EDTA buffer.

### Measurement of LINE-1 hypomethylation status

The hypomethylation status of LINE-1 was evaluated by a modified absolute quantitative analysis of methylated alleles (AQAMA) assay [12, 13, 16, 20]. In brief, AQAMA requires forward and reverse primers for amplifying a targeted sequence, methylation-specific and unmethylation-specific TaqMan probes with minor groove binder (MGB). The targeted sequence of LINE-1 was 148 bp in size and located in the promoter region of LINE-1. LINE-1 hypomethylation of this targeted region in early CRC tissue had already been confirmed in our previous study [12]. Primers and probes were purchased from Life Technologies. The following sets of primers were used: forward primer, 5′-GGGTTTATTTT ATTAGGGAGTGTTAGA-3′; reverse primer, 5′-TCAC CCCTTTCTTTA ACTCAAA-3′; methylation-specific probe, VIC-5′-TGCGCGAGTCGAAGT-3′-MGB; and unmethylation-specific probe, FAM-5′-TGTGTGAGT TGAAGTAGGG-3′-MGB. The AQAMA PCR reaction was performed with a 7500 Fast Real-Time PCR system (Life Technologies). All measurements were taken in triplicate of 20 μL consisting of 2 μL of bisulfite-converted DNA template, 10μL of KAPA PROBE FAST qPCR Master Mix (KAPA Biosystems), 0.4 μmol/L of the forward and reverse primer, and 0.25μmol/L of each MGB probe. The PCR conditions were as follows: 95°C for 3 min, followed by 40 cycles at 95°C for 15 s, and 60°C for 60 s. The absolute copy number of each sample was estimated from a standard curve with known copy numbers (10*6 to 10*1 copies).

The LINE-1 hypomethylation index (LHI) was defined as the unmethylated copy number/ (methylated copy number + unmethylated copy number), so that a greater LHI indicated enhanced LINE-1 hypomethylation. This LHI was used for the analysis.

### Construction of control plasmid

The universal unmethylated and methylated control DNA was synthesized from peripheral blood DNA of healthy donors using illustra GeomiPhi V2 DNA Amplification Kit (GE Healthcare) and M.SssI CpG methyltransferase (New England BioLabs), respectively. Both control DNA were bisulfite modified and were then amplified by PCR with the primer set of targeted region of LINE-1. The completely methylated and unmethylated PCR products were ligated into pCR 2.1-TOPO cloning vector (Life Technologies).

Methylated control plasmid was digested by Hind III and blunted with DNA Blunting Kit (TAKARA BIO), and then digested by Xho I. After the BamH I site of unmethylated control plasmid being destroyed by blunting with DNA Blunting Kit and self-ligation, unmethylated control plasmid was digested by EcoR V and Xho I. The Xho I and blunted Hind III fragment of methylated control DNA was ligated into the Xho I and EcoR V site of unmethylated control plasmid vector. Next the plasmid was linearized by BamH I digestion.

The constructed plasmid contained both methylated and unmethylated control sequences at the ratio of 1:1, and was PCR-amplified to make both methylated and unmethylated standard curves with methylation-specific and unmethylation-specific MGB probes in the same PCR well, enabling us to perform more accurate absolute quantification of methylated and unmethylated copy numbers.

### Statistical analysis

All analyses results presented in this study were based on biostatistical assessment. All statistical analyses were performed using JMP Pro version 10 software (SAS Institute). cfDNA concentration and cfDNA LHI were represented as the median with 95% confidence interval (CI), and were analyzed using Mann–Whitney U test (two groups) or Kruskal–Wallis test (multiple groups). The age and BMI of healthy donors and CRC patients were expressed as the means with SD, while Mann–Whitney U test was used for comparisons. Categorical variables were represented as numbers (%) and analyzed by Pearson's chi-squared test. In ROC curve analysis, the optimal cut-off value was defined as the highest Youden index [(specificity + sensitivity) − 1] [21]. Steel-Dwass test was performed for the nonparametric multiple comparison of cfDNA concentration and cfDNA LHI in healthy donors and CRC patients. Probability values (*P*) < 0.05 were considered statistically significant.

## References

[1] Brenner H, Kloor M, Pox CP (2014). Colorectal cancer. Lancet.

[2] Crowley E, Di Nicolantonio F, Loupakis F, Bardelli A (2013). Liquid biopsy: monitoring cancer-genetics in the blood. Nat Rev Clin Oncol.

[3] Heitzer E, Ulz P, Geigl JB (2015). Circulating tumor DNA as a liquid biopsy for cancer. Clin Chem.

[4] Schwarzenbach H, Hoon DS, Pantel K (2011). Cell-free nucleic acids as biomarkers in cancer patients. Nat Rev Cancer.

[5] Heyn H, Esteller M (2012). DNA methylation profiling in the clinic: applications and challenges. Nature Reviews Genetics.

[6] Elbarbary RA, Lucas BA, Maquat LE (2016). Retrotransposons as regulators of gene expression. Science.

[7] Cordaux R, Batzer MA (2009). The impact of retrotransposons on human genome evolution. Nat Rev Genet.

[8] Antelo M, Balaguer F, Shia J, Shen Y, Hur K, Moreira L, Cuatrecasas M, Bujanda L, Giraldez MD, Takahashi M, Cabanne A, Barugel ME (2012). A high degree of LINE-1 hypomethylation is a unique feature of early-onset colorectal cancer. PLoS One.

[9] Ogino S, Nosho K, Kirkner GJ, Kawasaki T, Chan AT, Schernhammer ES, Giovannucci EL, Fuchs CS (2008). A cohort study of tumoral LINE-1 hypomethylation and prognosis in colon cancer. J Natl Cancer Inst.

[10] Baba Y, Huttenhower C, Nosho K, Tanaka N, Shima K, Hazra A, Schernhammer ES, Hunter DJ, Giovannucci EL, Fuchs CS, Ogino S (2010). Epigenomic diversity of colorectal cancer indicated by LINE-1 methylation in a database of 869 tumors. Mol Cancer.

[11] Rhee YY, Kim MJ, Bae JM, Koh JM, Cho NY, Juhnn YS, Kim D, Kang GH (2012). Clinical outcomes of patients with microsatellite-unstable colorectal carcinomas depend on L1 methylation level. Ann Surg Oncol.

[12] Sunami E, de Maat M, Vu A, Turner RR, Hoon DS (2011). LINE-1 hypomethylation during primary colon cancer progression. PLoS One.

[13] van Hoesel AQ, van de Velde CJ, Kuppen PJ, Liefers GJ, Putter H, Sato Y, Elashoff DA, Turner RR, Shamonki JM, de Kruijf EM, van Nes JG, Giuliano AE (2012). Hypomethylation of LINE-1 in primary tumor has poor prognosis in young breast cancer patients: a retrospective cohort study. Breast Cancer Res Treat.

[14] Bae JM, Shin SH, Kwon HJ, Park SY, Kook MC, Kim YW, Cho NY, Kim N, Kim TY, Kim D, Kang GH (2012). ALU and LINE-1 hypomethylations in multistep gastric carcinogenesis and their prognostic implications. Int J Cancer.

[15] Shigaki H, Baba Y, Watanabe M, Murata A, Iwagami S, Miyake K, Ishimoto T, Iwatsuki M, Baba H (2013). LINE-1 hypomethylation in gastric cancer, detected by bisulfite pyrosequencing, is associated with poor prognosis. Gastric Cancer.

[16] Hoshimoto S, Kuo CT, Chong KK, Takeshima TL, Takei Y, Li MW, Huang SK, Sim MS, Morton DL, Hoon DS (2012). AIM1 and LINE-1 epigenetic aberrations in tumor and serum relate to melanoma progression and disease outcome. J Invest Dermatol.

[17] Harada K, Baba Y, Ishimoto T, Chikamoto A, Kosumi K, Hayashi H, Nitta H, Hashimoto D, Beppu T, Baba H (2015). LINE-1 methylation level and patient prognosis in a database of 208 hepatocellular carcinomas. Ann Surg Oncol.

[18] Benard A, van de Velde CJ, Lessard L, Putter H, Takeshima L, Kuppen PJ, Hoon DS (2013). Epigenetic status of LINE-1 predicts clinical outcome in early-stage rectal cancer. Br J Cancer.

[19] Mima K, Nowak JA, Qian ZR, Cao Y, Song M, Masugi Y, Shi Y, A da Silva, Gu M, Li W, Hamada T, Zhang X, Wu K (2016). Tumor LINE-1 methylation level and colorectal cancer location in relation to patient survival. Oncotarget.

[20] de Maat MF, Umetani N, Sunami E, Turner RR, Hoon DS (2007). Assessment of methylation events during colorectal tumor progression by absolute quantitative analysis of methylated alleles. Mol Cancer Res.

[21] Schisterman EF, Perkins NJ, Liu A, Bondell H (2005). Optimal cut-point and its corresponding Youden Index to discriminate individuals using pooled blood samples. Epidemiology.

[22] Pox CP, Altenhofen L, Brenner H, Theilmeier A, Von Stillfried D, Schmiegel W (2012). Efficacy of a nationwide screening colonoscopy program for colorectal cancer. Gastroenterology.

[23] Kuipers EJ, Rosch T, Bretthauer M (2013). Colorectal cancer screening--optimizing current strategies and new directions. Nat Rev Clin Oncol.

[24] Hirai HW, Tsoi KK, Chan JY, Wong SH, Ching JY, Wong MC, Wu JC, Chan FK, Sung JJ, Ng SC (2016). Systematic review with meta-analysis: faecal occult blood tests show lower colorectal cancer detection rates in the proximal colon in colonoscopy-verified diagnostic studies. Aliment Pharmacol Ther.

[25] Imperiale TF, Ransohoff DF, Itzkowitz SH, Levin TR, Lavin P, Lidgard GP, Ahlquist DA, Berger BM (2014). Multitarget stool DNA testing for colorectal-cancer screening. N Engl J Med.

[26] Chiu HM, Lee YC, Tu CH, Chen CC, Tseng PH, Liang JT, Shun CT, Lin JT, Wu MS (2013). Association between early stage colon neoplasms and false-negative results from the fecal immunochemical test. Clin Gastroenterol Hepatol.

[27] Smith RA, Andrews K, Brooks D, DeSantis CE, Fedewa SA, Lortet-Tieulent J, Manassaram-Baptiste D, Brawley OW, Wender RC (2016). Cancer screening in the United States, 2016: A review of current American Cancer Society guidelines and current issues in cancer screening. CA Cancer J Clin.

[28] von Wagner C, Baio G, Raine R, Snowball J, Morris S, Atkin W, Obichere A, Handley G, Logan RF, Rainbow S, Smith S, Halloran S (2011). Inequalities in participation in an organized national colorectal cancer screening programme: results from the first 2.6 million invitations in England. Int J Epidemiol.

[29] Taber JM, Aspinwall LG, Heichman KA, Kinney AY (2014). Preferences for blood-based colon cancer screening differ by race/ethnicity. Am J Health Behav.

[30] Tanaka K, Okamoto A (2007). Degradation of DNA by bisulfite treatment. Bioorg Med Chem Lett.

[31] Church TR, Wandell M, Lofton-Day C, Mongin SJ, Burger M, Payne SR, Castanos-Velez E, Blumenstein BA, Rosch T, Osborn N, Snover D, Day RW (2014). Prospective evaluation of methylated SEPT9 in plasma for detection of asymptomatic colorectal cancer. Gut.

[32] Nicholson BD, Shinkins B, Pathiraja I, Roberts NW, James TJ, Mallett S, Perera R, Primrose JN, Mant D (2015). Blood CEA levels for detecting recurrent colorectal cancer. Cochrane Database Syst Rev.

[33] Frattini M, Gallino G, Signoroni S, Balestra D, Lusa L, Battaglia L, Sozzi G, Bertario L, Leo E, Pilotti S, Pierotti MA (2008). Quantitative and qualitative characterization of plasma DNA identifies primary and recurrent colorectal cancer. Cancer Lett.

[34] Flamini E, Mercatali L, Nanni O, Calistri D, Nunziatini R, Zoli W, Rosetti P, Gardini N, Lattuneddu A, Verdecchia GM, Amadori D (2006). Free DNA and carcinoembryonic antigen serum levels: an important combination for diagnosis of colorectal cancer. Clin Cancer Res.

[35] Schwarzenbach H, Stoehlmacher J, Pantel K, Goekkurt E (2008). Detection and monitoring of cell-free DNA in blood of patients with colorectal cancer. Ann N Y Acad Sci.

[36] Danese E, Montagnana M, Minicozzi AM, De Matteis G, Scudo G, Salvagno GL, Cordiano C, Lippi G, Guidi GC (2010). Real-time polymerase chain reaction quantification of free DNA in serum of patients with polyps and colorectal cancers. Clin Chem Lab Med.

[37] Ogino S, Kawasaki T, Nosho K, Ohnishi M, Suemoto Y, Kirkner GJ, Fuchs CS (2008). LINE-1 hypomethylation is inversely associated with microsatellite instability and CpG island methylator phenotype in colorectal cancer. Int J Cancer.

[38] Matsunoki A, Kawakami K, Kotake M, Kaneko M, Kitamura H, Ooi A, Watanabe G, Minamoto T (2012). LINE-1 methylation shows little intra-patient heterogeneity in primary and synchronous metastatic colorectal cancer. BMC Cancer.

[39] Baba Y, Murata A, Watanabe M, Baba H (2014). Clinical implications of the LINE-1 methylation levels in patients with gastrointestinal cancer. Surg Today.

[40] Terry MB, Delgado-Cruzata L, Vin-Raviv N, Wu HC, Santella RM (2011). DNA methylation in white blood cells Association with risk factors in epidemiologic studies. Epigenetics.

[41] Hoon DS, Spugnardi M, Kuo C, Huang SK, Morton DL, Taback B (2004). Profiling epigenetic inactivation of tumor suppressor genes in tumors and plasma from cutaneous melanoma patients. Oncogene.

[42] Yamashita S, Takahashi S, McDonell N, Watanabe N, Niwa T, Hosoya K, Tsujino Y, Shirai T, Ushijima T (2008). Methylation silencing of transforming growth factor-beta receptor type II in rat prostate cancers. Cancer Res.

